# Navigating the future: horizon scanning and early dialogue in health technology assessment in Latin America

**DOI:** 10.1017/S0266462325100184

**Published:** 2025-07-10

**Authors:** Sebastián García Martí, Valentina Stacco, Andres Pichon-Riviere, Federico Augustovski, Andrea Alcaraz, Manuel A. Espinoza

**Affiliations:** 1 Institute for Clinical Effectiveness and Health Policy (IECS-CONICET), Buenos Aires, Argentina; 2 School of Public Health, School of Medicine, Pontificia Universidad Católica de Chile, Santiago, Chile

**Keywords:** horizon scanning, early dialogue, health technology assessment, Latin America, emerging technologies

## Abstract

**Objective:**

To systematize the information and perspectives shared during the 2024 LATAM policy forum, which explored advancements in horizon scanning and early dialogue processes in the region, by analyzing the main discussion and identifying the main lessons.

**Methods:**

This article is based on the discussions and background materials provided during the 1.5 days in-person 2024 Latin American Policy Forum (59 representatives from 11 countries). We gathered and systematized the information shared during the forum, including the results of a pre-forum survey. The Forum agenda included keynote presentations, breakout group activities, and plenary discussions to identify the main lessons and key messages from all different stakeholders’ points of view.

**Results:**

The forum highlighted the growing recognition of the need for structured horizon scanning and early dialogue processes in Latin America. Key barriers were identified, including the absence of clear legal frameworks, limited data availability, and the need for capacity-building. Potential solutions included fostering regional cooperation, improving transparency, and creating pilot programs for early engagement. Engaging patients and the pharmaceutical industry was deemed essential for trust and foster alignment between HTA agencies and regulators.

**Conclusions:**

Horizon scanning and early dialogue represent critical tools for improving health system preparedness and aligning innovation with local needs. Their implementation, however, requires coordinated efforts across multiple stakeholders, enhanced dialogue, and the development of supportive legal and regulatory frameworks.

## Introduction

The world is living in an unprecedented moment where technological innovation, population aging, and economic pressures overcome the capacity of health systems to provide timely access to health technologies. In this context, health technology assessment (HTA) (1) assists policy makers addressing the social, ethical, and legal implications of increasingly complex and costly healthcare systems to inform the incorporation of health technologies. The goal is to inform a fair, value-based adoption process while also exploring innovative methods and approaches that ensure timely and sustainable access to high-value technologies.

Incorporating new health technologies offers opportunities to improve service delivery and strengthen health systems care, management, and infrastructure, even during slow or stagnant economic growth (2). However, the rapid pace of technological advancement, combined with patient demand, has made it increasingly difficult for health systems to plan and manage these innovations effectively.

Horizon scanning is a systematic process for identifying emerging health technologies that may pose opportunities, risks, or challenges to healthcare systems (3). It supports policy makers, purchasers, and healthcare providers by informing research prioritization, financial and operational planning, and the controlled diffusion of technologies to ensure early yet responsible access. Furthermore, it may include health technologies that are becoming obsolete and that have the potential to affect health, health services, and/or society. Horizon scanning and early dialogue have grown in popularity across countries with HTA processes, by contributing to each system’s preparedness for new technologies. These approaches enable health systems to anticipate and align technological innovations with healthcare needs (4).

Early dialogue refers to a structured, collaborative process between healthcare technology developers, regulatory bodies, and HTA agencies to align interests between developers and health systems during the early stages of technology development. These processes provide an opportunity to ensure that new technologies meet health system needs upon their introduction, promoting an appropriate research plan and forecast for the development of pharmaceuticals, medical devices, and diagnostics (5).

Although proactive planning is essential for ensuring universal access to new technologies while maintaining financial and organizational sustainability (3), horizon scanning and early dialogue remain underdeveloped in Latin America. Health systems in the region are often fragmented, with varying levels of HTA maturity, and many countries still rely on *ad hoc* or informal mechanisms to address these processes.

Recognizing these challenges, the IX HTAi Latin American Policy Forum, held in Cartagena de Indias, Colombia, in August 2024, focused on the relevance of horizon scanning and early dialogue for the region. Each year, the Policy Forum selects a topic collaboratively among its members, and in 2024, these themes were prioritized due to the limited knowledge about their implementation and potential impact among key stakeholders of the LATAM PF.

## Methods

The 2024 HTAi LATAM Policy Forum brought together stakeholders from patient organizations, HTA agencies, payers, and the pharmaceutical industry for a 1.5-day in-person meeting (see Supplementary Annex I for the full participant list).

To ensure balanced discussions, preparatory materials were shared in advance, and the agenda was structured with keynote presentations, breakout sessions, and plenary discussion (see Table 1. Session structure of the 2024 HTAi Latin American Policy Forum). Discussion focused on the challenges and opportunities of implementing horizon scanning and early dialogue in Latin America, drawing on international models and regional perspectives. A background paper provided (6) key definitions, case studies, and global insights to align terminology and support meaningful dialogue.

**Table 1.** Session structure of the 2024 HTAi Latin American Policy Forum

Stage	Focus
Opening and keynote address	Inaugural session introducing key themes, delivered by a renowned expert voice in the field.
Panel discussions	Expert discussions on regional and global perspectives
Breakout group sessions (day 1 – Horizon scanning)	Interactive sessions on smaller groups focusing on horizon scanning
Plenary discussion	Reflection and sharing with all participants
Summary of key takeaways from day 1	Review and analysis on the main points from the previous day
Breakout group sessions (day 2 – Early dialogue)	Specific sessions on smaller groups focusing on early dialogue
Final plenary reflection and concluding remarks	Final discussion summarizing the forum’s conclusions

Ahead of the meeting, a survey was conducted among participants from 11 countries to assess the current use of these mechanisms, providing insights into their implementation and challenges across the region.

On the first day, horizon scanning and early dialogue were introduced as topics. Their integration into HTA processes was discussed from both, international and regional perspectives, and different stakeholders, with different viewpoints shared by panelists from the drugs and devices industry, as well as a patient representative. Experiences from Brazil and Colombia were presented as case studies.

Breakout group activities were divided into thematic areas – horizon scanning and early dialogue – structured to foster discussion on their feasibility within Latin American health systems. The breakout group activities were carried out based on a discussion-and-debate methodology, with practical processes to redefine problems and find solutions. Participants, representing diverse countries and sectors, engaged in a structured debate methodology to redefine challenges and propose solutions. Over the two days, discussions focused on:

- Day 1: Assessing the necessary information and feasibility of implementing horizon scanning in the region.
- Day 2: Evaluating different stages and objectives for implementing early dialogue.

Plenary sessions analyzed key takeaways from these discussions, offering valuable insights to guide the integration of these processes in Latin America.

This report summarizes the main discussion points and lessons from the deliberative exercise. The Forum was conducted under Chatham House Rules (7), and some concepts from the design thinking methodology were incorporated in the group activities (8).

## Results

The IX HTAi LATAM Policy Forum gathered 59 participants, including representatives from HTA agencies, industry, patient organizations, and international bodies from 11 Latin American countries: Argentina, Brazil, Chile, Colombia, Costa Rica, the Dominican Republic, Ecuador, El Salvador, Mexico, Peru, and Uruguay.

### Potential benefits and implementation phases in horizon scanning and early dialogues processes in the region

Participants agreed on the potential benefits of horizon scanning and early dialogue for Latin American health systems:

Horizon scanning was seen as valuable for:

- Anticipating the arrival of new technologies while phasing out those no longer in use.
- Informing decision making to support the efficient allocation of healthcare resources.

Early dialogue facilitates:

- Stakeholder alignment, including technology developers, regulators, and HTA agencies.
- Optimization of technology development and approval processes.
- Adaptation of research and technology development to regional needs.

**Figure 1.**
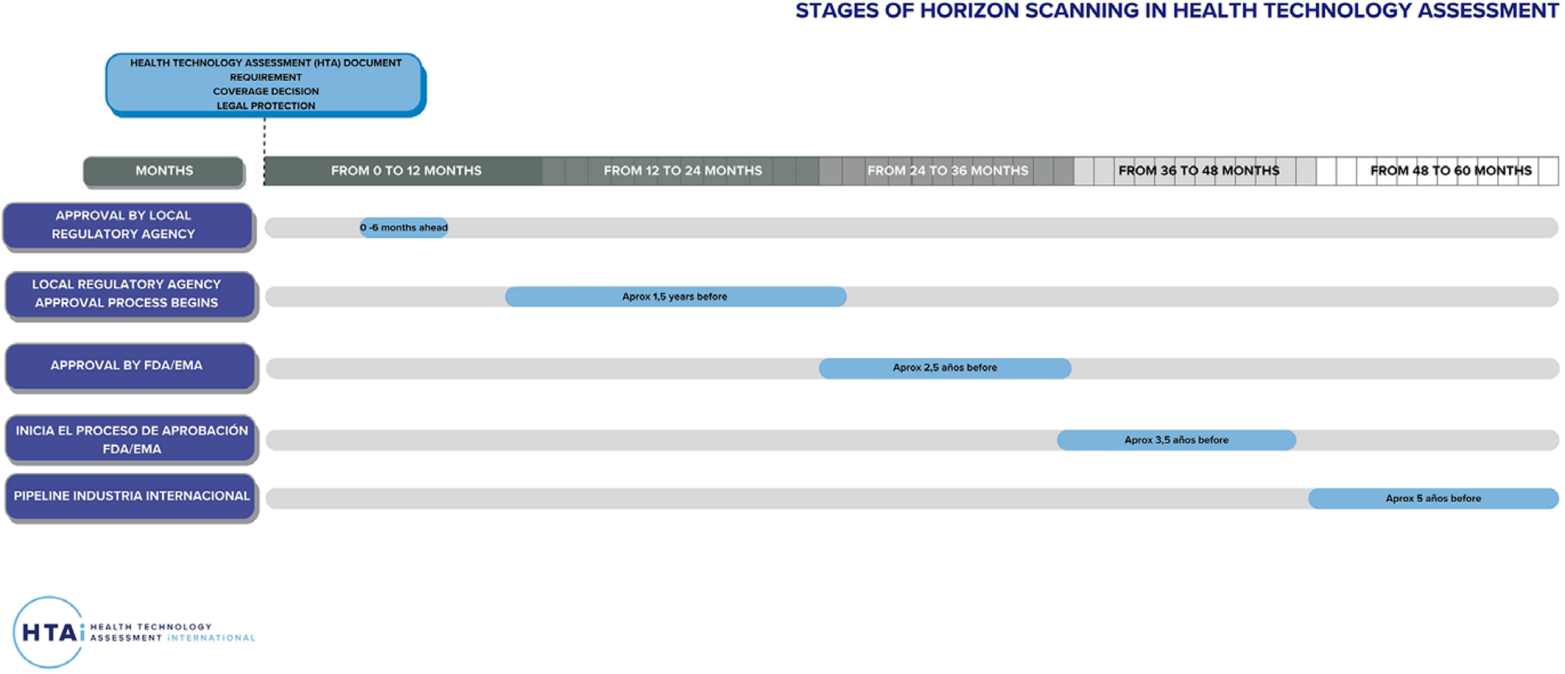
Stages of Horizon Scanning in Health Technology Assessment.

During the Forum, a structured horizon scanning process was discussed (Figure 1. HTA horizon scanning stages), outlining key phases from the identification of technologies in global pipelines (~5 years before approval) to final national regulatory decisions. Participants prioritized two phases as most relevant for the region:

- Approval by international regulatory agencies (FDA/EMA) (~3.5 years before market entry).
- Initiation of local regulatory approval processes.
- During the group sessions, participants proposed incorporating another distinct instance as a “state alarm” in the horizon scanning phases scheme to address unforeseen health crises (e.g., COVID-19) and enable a rapid response and search for solutions. Table 2 summarizes and systematizes the discussion regarding each phase that were raised in the plenary session. Regarding early dialogue, a number of modalities were suggested as types of interactions that take place between stakeholders and were validated during the breakout group activities. Each of these modalities involves at least two stakeholders. From the health system’s perspective, these may include government institutions, scientific societies, civil society organizations, and patient groups, with the possibility of multiple stakeholders participating simultaneously. These modalities are presented as follows. With technology, producers outline potential research directions based on the priorities and needs of the health system at very early stages, even before specific technologies exist.
- With technology, producers define methodological aspects of studies in the early phases of clinical research (comparators, outcomes, etc.) prior to regulatory approval.
- Between technology producers and regulatory agencies to align information requirements for regulatory approval submissions.
- Between HTA agencies and technology producers to coordinate submissions for HTA evaluations following regulatory approval.
- Between regulatory and HTA agencies to harmonize information requirements and streamline approval processes.

**Table 2.** Key decisions and actions informed by HTA horizon scanning across phases

Phase	Timeframe	Decisions and actions
Local regulatory agency approval	0–6 months prior	Early HTA planning (e.g., comparator selection); financial planning (disinvesting); patient and staff training; infrastructure and care model redesign; clinical guidelines adaptation; price negotiation considerations; minister of health prioritization support
Start of local regulatory process	~1.5 years prior	Proactive HTA prioritization; innovation fund establishment; anticipation of legislative changes; healthcare workforce training; reduced approval time for priority technologies; evaluation of local evidence gaps; risk-sharing agreements preparation
FDA/EMA approval	~2.5 years prior	Clinical guidelines updates; therapeutic area prioritization; comparator definition; budget planning; local disease burden data generation
Start of FDA/EMA approval process	~3.5 years prior	Financial impact assessments; collaboration with technology producers; risk-sharing program preparation; infrastructure and workforce upgrades; regional research planning
Industry pipeline	~5 years prior	Technology identification for transfer negotiations^a^; resource allocation for data generation; supply chain preparation; ongoing value framework reassessment

a Technology assessment before transfer agreement negotiations, including licensing/commercialization terms.

The “technology producers” category includes both small start-ups and academic units developing initial ideas, as well as “Big Pharma.” During the plenary session, an electronic voting platform was used to prioritize instances where the region could implement early dialogue activities, prioritizing those closest to coverage decisions, such as coordination between regulatory and HTA agencies. Earlier-stage dialogues (e.g., clinical study design discussions) were considered lower priority.

In the context of early dialogue, several objectives were identified as potentially beneficial for the region, illustrating the outcomes that can be achieved during each phase of the process. A summary is presented in Tables 3 and 4.

**Table 3.** Key aspects for promoting horizon scanning

Topic	Concept
Redefinition of health	Emphasizing health’s social dimensions within value frameworks, considering broader societal impacts
Budget dynamics	Enabling budget reallocation to improve flexibility and efficiency, rather than rigid fund definitions
HTA agency involvement	Expanding the scope of HTA agencies was seen as beneficial for health system decisions
Technology focus	Focusing on regular cycles to update benefits plans, not just individual technologies
Price negotiation	Keeping HTA evaluations evidence-based and separate from financial negotiations
Agency alignment and stakeholder engagement	Aligning HTA and regulatory bodies, engaging stakeholders early in the process
Reliance concept	Leveraging international decisions to accelerate availability and reduce duplication
Legislative adaptation	Adapting regulatory frameworks to keep pace with technological innovation

**Table 4.** Contributions and impact of early dialogue implementation across different phases

Phase	Objectives
Initial phase prior to technology existence	Align development with health system priorities; assess capacity; define LATAM-specific objectives; develop local investment strategies
Definition of methodological aspects for early studies	Identify valid evidence sources (e.g., RWE); define PICO; ensure regulatory guideline availability; agree on risk plans; adapt trials to local needs; encourage local partnerships
Alignment for regulatory approval	Plan for local participation; identify expedited processes; enable access for compassionate use
Alignment for HTA submission	Streamline approval; reduce uncertainty; hold pre-submission meetings; align evidence expectations; map civil society needs; improve submission quality
Optimize approval between regulatory and HTA agencies	Facilitate access; prioritize HTA after regulatory approval; assess HR needs; plan for sustainable supply

### Prioritization criteria to select technologies to evaluate the potential impact on health systems

As previously mentioned, horizon scanning is a strategic process designed to identify, filter, and prioritize emerging technologies with the potential to impact healthcare systems (4). In this context, forum participants agreed that focusing horizon scanning efforts on technologies already approved by the U.S. Food and Drug Administration (FDA) or the European Medicines Agency (EMA) is more convenient for the region than targeting those in earlier development phases. Given the vast range of possible health technologies, it is crucial to select only those innovations likely to have the highest impact, thereby enabling healthcare agencies to allocate resources efficiently, ensure that only the most promising options undergo in-depth HTA (4), and prioritize local health needs over merely reacting to international trends. A roundtable discussion identified and ranked the most relevant criteria for selecting high-impact technologies:

- High clinical impact/disruptive or transformative technologies.
- Relevance to local health needs and priorities.
- High budget impact.
- High burden of disease.

Criteria related to the burden of disease are more relevant during the pipeline evaluation stage, while those concerning efficacy align more closely with regulatory processes.

Other relevant factors included population size, media impact, political priority, and lack of alternative treatments.

The link between technology prioritization and equity was stressed, emphasizing the importance of involving both patients and clinicians. By incorporating these complementary perspectives, the approach fosters a more equitable health system, ensuring that innovation benefits individuals, caregivers, and communities comprehensively. Potential sources for identifying the range of technologies that can affect health systems were considered valid and legitimate for gathering information, including hospital records, clinical trial databases, epidemiological reports, scientific publications, and industry websites. Participants also discussed the importance of leveraging existing horizon scanning agencies (e.g., EuroScan), which can help reduce the manual search burden and facilitate the early identification of impactful technologies.

### Challenges in adopting horizon scanning and early dialogue across the region

A preforum survey (see Supplementary Annex II for results in English) gathered 35 responses from nine countries, revealing key barriers to implementation of these processes. The survey found that 50 percent of respondents neither conduct nor are familiar with horizon scanning, and seventy-five percent reported the same for early dialogue. A lack of awareness, political will, and funding constraints were identified as the primary barriers. Additionally, technical skill gaps, distrust among stakeholders, and a perception that these tools are irrelevant to local contexts further hindered adoption. Participants also emphasized the need for evaluation frameworks to promote transparency and trust between industry and regulators.

Discussions during the forum identified additional obstacles: the absence of dedicated teams, inadequate guidelines and frameworks, and limited institutional capacity. Also, restricted public access to information – exemplified by BRISA (BRISA – RedETSA), the regional HTA database – further impedes progress. Although BRISA houses 3,500 HTA reports, only two are related to horizon scanning, underscoring the under-prioritization of this tool.

A regional panel offered insights into how Brazil and Colombia are navigating these challenges; both countries report progress, yet horizon scanning and early dialogue in Latin America remain underdeveloped due to resource constraints, limited technical capacity, and fragmented health systems.

Pharmaceutical representatives at the forum stressed the need for stronger collaboration to expedite patient access to innovative treatments. They highlighted that trust among stakeholders is essential, with the industry potentially collaborating by offering information to support evaluation efforts.

Patient organizations underscored the importance of empowering patients to participate actively in the evaluation and prioritization of health technologies. They emphasized that evaluations should capture not only patient’s quality of life but also the well-being of caregivers. While Colombia’s Ley Estatutaria 1751 (Ley 1751 de 2015 - Gestor Normativo) promotes patient participation in healthcare decisions, consistent implementation and meaningful involvement remain challenges.

During breakout group sessions, participants identified critical barriers to early dialogue implementation. In the plenary, participants voted using an electronic polling app, the top challenges identified were:

- Lack of guidelines and frameworks;
- Lack of trust among stakeholders;
- Limited institutional capacity;
- Insufficient technical training, particularly in economic evaluations.

These discussions highlighted that, despite the promise of horizon scanning and early dialogue, their effective implementation requires addressing structural barriers and fostering collaboration across sectors.

### Integrating early dialogue and horizon scanning into health technology assessment processes

Drawing from international experiences, a senior expert from the U.K.’s National Institute for Health Research (NIHR) presented horizon scanning as a key component of HTA. Over three decades of experience with technologies nearing market entry have demonstrated the importance of setting clear objectives, establishing structured processes, selecting appropriate technologies, and measuring impact. This is crucial to align innovations with healthcare priorities effectively.

During the first panel, international experts shared their previous experiences and provided recommendations for Latin America, emphasizing the role of early dialogue in aligning clinical research with both market and regulatory needs, even before phase III trials. They discussed the importance of shifting from reactive to proactive approaches in technology evaluation, demonstrating how horizon scanning has enhanced healthcare delivery.

Early dialogue was said to contribute to different stages in the technology lifecycle, including early alignment between developers and health systems, methodological discussions during clinical studies, and pre-submission consultations with HTA agencies. The experts stressed the need for transparency, stakeholder engagement, and alignment between regulatory and HTA requirements to streamline approvals and improve evaluations. Their recommendations for Latin America focused on adopting scientific dialogue to engage stakeholders, accounting for the specific characteristics of the region’s health systems, and implementing proactive horizon scanning to identify needs early and establish clear guidelines. Additionally, they highlighted the importance of further research to tailor these processes to the regional context and develop suitable frameworks and guidelines for effective implementation.

Participants also emphasized horizon scanning should extend beyond individual technologies to encompass public health, telemedicine, and artificial intelligence (AI). This broader scope would help manage regulatory changes, economic shifts, and innovations with significant societal impact. Government involvement – through economy, education, and science ministries – was seen as a way to enable flexible budget allocations based on cost-effectiveness and anticipated improvements.

A senior representative from the International Horizon Scanning Initiative (IHSI) presented the organization’s work, focusing on forecasting future pharmaceutical products through a database populated with public data from European sources. The IHSI emphasized its transparency and independence, being fully funded by national governments. The database aggregates information from research institutes, industries, and the stock market.

A leading expert from Malaysia shared their experience integrating horizon scanning into their HTA agency, explaining their systematic methodology and the impact of horizon scanning reports on improving decision-making processes.

Participants emphasized the importance of strengthening trust, including improving transparency in decision-making processes, actively engaging stakeholders throughout horizon scanning and early dialogue, and involving patients, payers, and manufacturers in discussions about the risks and benefits of new technologies.

### Collaborative strategies to facilitate adoption

Implementing horizon scanning and early dialogue presents significant challenges, among them the lack of appropriate guidelines and frameworks for conducting dialogue, limited trust among stakeholders, and the substantial resource demands required.

Therefore, there was broad agreement on the importance of ongoing collaboration and capacity-building to support their adoption. In contexts with limited financial and technical resources, pooling expertise and resources across countries is crucial to strengthen health systems and avoid duplication. Regional cooperation, facilitated by multilateral organizations like the Pan American Health Organization (PAHO), was identified as essential for improving data sharing and developing shared frameworks to integrate horizon scanning and early dialogue.

In this respect, the EuroScan (9) International Network illustrates how collaborative horizon scanning efforts can enhance health systems’ capacity to adopt emerging innovations. As a global consortium of publicly funded agencies, EuroScan concentrates on the early identification and assessment of health technologies while promoting information exchange and expertise-sharing among its members. This model addresses key factors such as safety, efficacy, and strategic planning, ultimately bolstering preparedness for the adoption of new innovations.

To explore the feasibility of horizon scanning and early dialogue in the region, participants proposed implementing pilot projects focused on specific technologies or sectors. These pilots could generate valuable insights into the practical challenges involved in integrating horizon scanning and early dialogue into the broader HTA framework. Likewise, there was consensus on the importance of collaboration among various stakeholders, including ETESA agencies, regulatory bodies, industry, and patients, to ensure a comprehensive approach. The Netherlands’ experience with parallel scientific advice, along with other European initiatives, serves as a reference for aligning regulatory and reimbursement expectations through early cooperation among regulatory agencies, HTA bodies, technology producers, and patients. This approach supports more efficient product development by offering simultaneous feedback, helping to minimize evidence misalignments and improve coordination among stakeholders. While the model has shown potential in preventing market access delays, its effectiveness varies depending on specific contexts, providing useful insights for regions looking to refine decision-making frameworks. As previously mentioned, the Forum underscored the significance of patient involvement, not only in the prioritization of health technologies but also in evaluating their impact on quality of life and alignment with their needs.

## Conclusions – Key messages

The 2024 HTAi Latin American Policy Forum highlighted both the potential opportunities and challenges of implementing horizon scanning and early dialogue in the region. Both tools were recognized as essential for improving evaluation processes and making them more efficient.

The forum identified well-organized international experiences, such as the initiative led by the Innovation Observatory, which serves as the national horizon scanning entity for NIHR. This approach ensures that ninety-five percent of the U.K.’s horizon scanning is publicly accessible. Additionally, consolidated and inclusive networks providing valuable opportunities for countries to enhance collaboration were addressed. For instance, IHS offers one through government-sponsored memberships.

Meanwhile, horizon scanning systems exhibit significant variability, with no single method or guideline universally defining what content should be collected, analyzed, or reported. Moreover, horizon reports are not standardized as their structure has not yet been convened. As a result, countries face the challenge of making their own pathway by developing systems from scratch, with the methodological and conceptual inconveniences this may pose. Despite this, the forum raised a number of similarities. Various stages of its execution were identified and prioritized, from approval by the local regulatory agency to evaluating the industry pipeline. The intermediate stages (FDA/EMA approval) were considered a priority for the region, over those that are imminent (approval by the regulatory agency) or distant (international industry pipeline). A particular consideration for the region concerning these processes is the temporal difference regarding the introduction of technologies into regional health systems compared to other places in the world. Sometimes, by the time technologies arrive in the region, they have already been approved by FDA/EMA, which makes the approach to horizon scanning processes in the region different. This also alters the objectives of early dialogue instances, for example, regarding the interaction between technology developments and the local needs of regional systems. In general, the region is not searching in the distant future, but attending to closer instances; nearer to the technology incorporation. It seems that, in general, in the region, “horizon scanning is not so far, and early dialogue is not so early.” Participants agreed that focusing horizon scanning efforts on technologies already approved by the FDA or EMA would be more effective than targeting those in early phases, ensuring that evaluations are timely and aligned with the technologies most likely to enter the region in less time.

However, several barriers must be addressed, including the absence of adequate guidelines and frameworks, limited institutional capacity, technical skill gaps, distrust among stakeholders, and restricted public access to information. Thus, it is clear that discussions and agreements still remain unexplored, as well as fostering trust among stakeholders. To a large extent, this is due to a lack of clear terms of reference and specific requirements to be fulfilled by the different entities involved in each process and stage.

Another topic raised during the forum was the need to adapt early dialogue and horizon scanning to the specific realities of the region, taking into account local priorities and the capacity of regional health systems. Unlike conventional approaches used globally, Latin America’s limited resources highlight the importance of tailoring these strategies to address regional needs effectively, ensuring a more equitable and efficient adoption of new health technologies. One of the relevant outputs of the forum was a key prioritization criteria that could help classify those new and emerging technologies that would encompass a greater impact on health systems in the region. The filter included aspects such as clinical impact, alignment of the technology with local needs, budget impact, and disease burden.

To improve the integration of horizon scanning and early dialogue, participants recommended establishing legal frameworks, strengthening institutional capacity through targeted training (especially in economic evaluations), and enhancing regional cooperation. Pilot programs focused on specific technologies were proposed as practical ways to test these tools, together with a greater patient and pharmaceutical industry engagement that was seen as critical to building trust and aligning expectations across sectors.

Future collaborative initiatives are needed to develop optimal frameworks tailored to the region’s specific context, measure the impact of these tools on decision making, and promote collaboration between regulatory bodies and HTA agencies. Future studies should also explore ways to engage patients meaningfully in these processes and assess the broader applications of horizon scanning and early dialogue.

## Supporting information

García Martí et al. supplementary material García Martí et al. supplementary material

## Supplementary material

The supplementary material for this article can be found at http://doi.org/10.1017/S0266462325100184.
