# Supplementary material for: Navigating the future: horizon scanning and early dialogue in health technology assessment in Latin America
Source: Int J Technol Assess Health Care. 2025 Jul 10;41(1):e42. doi: 10.1017/S0266462325100184 (PMC12257038; doi:10.1017/S0266462325100184)
Supplement: García Martí et al. supplementary material [file S0266462325100184sup001.docx]

# **ANNEX I**

## List of attendees of the IX Latin American Forum on Health Technology Assessment Policies

We would like to thank all participants for their interest, engagement, and contributions:

### **Industry Participants**

1. Diego Guarín, Executive Director, Regional Market Access, Merck (MSD) / United States
2. Mohit Jain, VP Global Head, Value, Access, and Strategic Pricing, BioMarin Pharmaceutical Inc / UK
3. Carlos Kane, Director of Market Access and Value Proposition, Eli Lilly / Mexico
4. Diego Kanevsky, Manager of Health Economics and Outcomes Research, Abbvie / Argentina
5. Arely Lemus Carmona, Value Access Director, BMS / United States
6. Kariluz Maestre, LATAM Market Access Implementation Lead, Astellas / Colombia
7. Vitor Nasciben, Senior Director of LATAM Market Access, Johnson and Johnson / United States
8. Fabian Ochoa Rubio, Market Access, Edwards Lifesciences / Colombia
9. Melanie Paccot, Director of Value, Access, and Trade, Novartis / Chile
10. Cintia Parellada, Executive Director of Research Outcomes, MSD / Brazil
11. Ricardo Salazar, Director of LATAM Market Access and Government Affairs, Abbvie / Colombia
12. Antonio Santos, Global Director of Medical Affairs in Rare Hematology, Sanofi / Brazil
13. Jose Thomaz, Senior Director of Market Access, Intercontinental Region, BioMarin Pharmaceutical Inc / Brazil
14. Atanacio Valencia, Director of Health Economics, Pricing, and RWE, Johnson and Johnson / Mexico
15. Joice Valentim, Global Strategy Director in HTA, F. Hoffmann-La Roche / Switzerland
16. Diana Carolina Díaz, Director of Corporate Affairs, Eli Lilly / Colombia
17. Antonio Acosta Aguirre, Associate Access Director for Colombia and Peru, BMS / Colombia
18. Marcia Alvez, Senior Director of Public Affairs, Edwards Lifesciences / Brazil
19. Juan Guillermo Ariza, Health Economics and Outcomes Research Lead, International Markets, Astellas / Colombia
20. Virginia Becerra, Access Director for Uruguay, Bolivia, and Paraguay, Sponsor of the Latin American HTA Network, Roche / Uruguay
21. Artur Brito, Manager of Public Strategies, Novartis / Brazil
22. Alicia Granados, Head of Global Medical-Scientific Advocacy for Rare Diseases, Sanofi / Spain

### **Participants from Ministries of Health, healthcare technology agencies, payers**

1. Andrea Guerrero Ahumada, Department Director, Ministry of Health / Chile
2. Santiago Hasdeu, Executive Coordinator, RedARETS / Argentina
3. Alvaro Hernandez Caballero, Director of the Medications Area, Mexican Social Security Institute (IMSS) / Mexico
4. Hugo Marín Piva, Director of the Pharmacoeconomics Area, Costa Rican Social Security Fund / Costa Rica
5. Madeline Martinez, Director of the Department of Health Technology Assessment and Economic Analysis, Superintendence of Health and Occupational Risks / Dominican Republic
6. Andrea Brígida de Souza, Coordinator, CONITEC / Brazil
7. Graciela Fernandez, Deputy Technical-Medical Director, National Resource Fund / Uruguay
8. Hellen Miyamoto, Superintendent, National Federation of Health Insurance / Brazil
9. Adriana Robayo, Director of IETS / Colombia
10. Ana Eduviges Sancho Jimenez, Head of the Health Technologies Unit, Ministry of Health / Costa Rica
11. Raul Alonso Timana Ruiz, Director, National Institute of Health / Peru
12. Felipe Vera Chandia, Advisor to the Ministry of Health / Chile
13. Lizbeth Acuña Merchán, Executive Director of the High-Cost Account / Colombia
14. Leonardo Basso, Financial Manager, AETSU / Uruguay
15. Silvina Benchetrit, Operations Manager, Ministry of Health, City of Buenos Aires / Argentina
16. Daniela Astrid Calderon Sanchez, Head Medications Section, Salvadoran Social Security Institute / El Salvador
17. María Jose Falconi, National Director of Health Technology Assessment, Ministry of Public Health / Ecuador
18. Luz Mery Gonzales, Deputy Director, Institute of Health Technology Assessment (IETS) / Colombia

### **Patients Representatives**

1. Diego Fernando Gil Cardozo, Executive Director, Colombian Federation of Rare Diseases / Colombia
2. Brayhan Yesid González Gutierrez, Legal Representative, Pompe Club International Foundation / Colombia
3. Eva María Ruiz de Castilla, Executive Director, Latin America Patients Academy / United States
4. Luz Victoria Salazar Ceballos, Executive Director, ACOPEL / Colombia

### **Speakers and Panelists**

1. Dawn Craig, Professor, New Castle University / United Kingdom
2. Alicia Granados, Head of Global Medical-Scientific Advocacy for Rare Diseases, SANOFI / Spain
3. Wija Oortwijn, Former President of HTAi, RadboudUMC / Netherlands
4. Iñaki Gutiérrez-Ibarlucea, Director of Knowledge, Management and Evaluation, Bioef-Osteba / Spain
5. Izzuna Mudla Mohamed Ghazali, Head of HTA Section, Ministry of Health / Malaysia
6. Marcus Guardian, General Manager, International Horizon Scanning Initiative (IHSI) / Belgium
7. Vania Canuto, Technical Officer, Pan American Health Organization / Brazil

### **HTAi Authorities**

1. Rabia Sucu, President of HTAi / United States
2. Ann Single, Vice President of HTAi / Australia
3. Manuel Espinoza, Chair, HTA Latin American HTA Policy Forum / Chile

### **HTAi - Logistics and Meeting planners**

1. Alicia Powers / Coordinator Events Manager, Health Technologies Assessment International (HTAI) / Canadá
2. Hana Price / Events Manager, Health Technologies Assessment International (HTAI) / Canadá

### **IECS (Scientific Secretariat)**

1. Andrés Pichón-Riviere, Scientific Secretariat, Instituto de Efectividad Clínica y Sanitaria / Argentina
2. Sebastián García Martí, Scientific Secretariat, Instituto de Efectividad Clínica y Sanitaria / Argentina
3. Federico Augustovski, Scientific Secretariat, Instituto de Efectividad Clínica y Sanitaria / Argentina
4. Valentina Stacco, Scientific Secretariat, Instituto de Efectividad Clínica y Sanitaria / Argentina
5. Andrea Alcaraz, Scientific Secretariat, Instituto de Efectividad Clínica y Sanitaria / Argentina

## Annex II

### **Survey of current situation in the region -- Results**

**Q1. Please state your institutional affiliation**

Respondents: 35

- Technology producers – 42.86 percent (15)
- Patient representatives – 5.71 percent (2)
- Government agency – 28.57 percent (10)
- Payer – 14.29 percent (5)
- Academia – 11.43 percent (4)
- Pan American Health Organization – 2.86 percent (1)
- Other (please specify) – 5.71 percent (2)

**Q2. Please specify your country of origin**

**Respondents: 35**

- Argentina – 14.29 percent (5)
- Brazil – 22.86 percent (8)
- Costa Rica – 5.71 percent(2)
- Chile – 8.57 percent (3)
- Colombia – 17.14 percent (6)
- Ecuador – 2.86 percent (1)
- Uruguay – 5.71 percent (2)
- Dominican Republic – 2.86 percent (1)
- Jamaica – 0.00 percent (0)
- Mexico – 5.71 percent (2)
- Peru – 0.00 percent (0)
- El Salvador – 0.00 percent (0)
- Other (please specify) – 14.29 percent (5)

**Q3. Do you consider that in your country or the countries you work in, there are activities related to Horizon Scanning (HS)?**

**Respondents: 35**

- Yes – 45.71 percent (16)
- No – 51.43 percent (18)
- Don’t know – 2.86 percent (1)

**Q4. What level of HS implementation exists in your country (1–5)?**

***(1: non-systematic and very occasional; 5: fully institutionalized)* Respondents: 15**

- 1 – Very occasional – 6.67 percent (1)
- 2 – Occasional – 40.00 percent (6)
- 3 – Regular and somewhat systematic – 26.67 percent (4)
- 4 – Systematic and frequent – 13.33 percent (2)
- 5 – Fully institutionalized – 13.33 percent (2)

**Q5. How far in advance do you consider HS is conducted before HTA evaluation?**

**Respondents: 15**

- More than 4 years – 20.00 percent (3)
- Between 3 and 4 years – 6.67 percent (1)
- Between 2 and 3 years – 26.67 percent (4)
- Between 1 and 2 years – 26.67 percent (4)
- Less than 1 year – 20.00 percent (3)

**Q6. Are there explicit criteria used to identify, select, and prioritize technologies for HS?**

**Respondents: 15**

- Yes – 26.67 percent (4)
- No – 53.33 percent (8)
- Don’t know – 20.00 percent(3)

**Q7. Are there methodological guides or formal processes for identifying and evaluating interventions for HS?**

**Respondents: 14**

- Yes – 35.71 percent(5)
- No – 42.86 percent (6)
- Don’t know – 21.43 percent (3)

**Q8. Who is responsible for HS activities in your country? (multiple choice)**

**Respondents: 13**

- Ministry of Health – 7.69 percent (1)
- HTA agency – 92.31 percent (12)
- Academic institution – 23.08 percent (3)
- Technology producer – 23.08 percent (3)
- Other (specify) – 7.69 percent (1)

**Q9. Is early dialogue conducted?
Respondents: 15**

- Yes – 58.33 percent (7)
- No – 41.67 percent (5)

**Q10. Which modalities are used? *(Multiple responses allowed)*
Respondents: 12 *(only those who conduct early dialogue)***

- Informal consultations – 72.00 percent (9)
- Structured scientific advice – 38.00 percent (5)
- Multi-stakeholder workshops/forums – 25.00 percent (3)

**Q11. Level of strength in implementation
Respondents: 15**

- Fully institutionalized – 13.33 percent (2)
- Partially implemented/pilot phase – 46.67 percent (7)
- Informal or ad-hoc only – 40.00 percent (6)

**Q12. Barriers to early dialogue *(Multiple responses allowed)*
Respondents: 15**

- Lack of awareness of potential benefits – 60.00 percent (9)
- Lack of trust among stakeholders – 46.67 percent (7)
- Lack of technical skills – 40.00 percent (6)
- Budget constraints – 53.33 percent (8)
- The need is not yet fully recognized – 33.33 percent(5)
- Concerns over conflicts of interest – 26.67 percent(4)

**Q13. Potential objectives of early dialogue *(Multiple responses allowed)*
Respondents: 15**

- Aligning regulatory and HTA requirements – 66.67 percent (10)
- Defining methodological aspects of clinical studies – 60.00 percent (9)
- Identifying research priorities – 53.33 percent (8)

**Q14. The usefulness of early dialogue
Respondents: 15**

- Very useful – 33.33 percent (5)
- Somewhat useful – 46.67 percent (7)
- Not useful – 13.33 percent (2)
- Don’t know – 6.67 percent (1)
